# An efficient ensemble method for missing value imputation in microarray gene expression data

**DOI:** 10.1186/s12859-021-04109-4

**Published:** 2021-04-13

**Authors:** Xinshan Zhu, Jiayu Wang, Biao Sun, Chao Ren, Ting Yang, Jie Ding

**Affiliations:** 1 School of Electrical and Information Engineering, Tianjin University, Tianjin, 300072 China; 2 State Key Laboratory of Digital Publishing Technology, Beijing, 100871 China; 3 China Institute of FTZ Supply Chain, Shanghai Maritime University, Shanghai, 201306 China

**Keywords:** Gene expression data, Imputation, Ensemble learning, Bootstrap sampling, Generalization

## Abstract

**Background:**

The genomics data analysis has been widely used to study disease genes and drug targets. However, the existence of missing values in genomics datasets poses a significant problem, which severely hinders the use of genomics data. Current imputation methods based on a single learner often explores less known genomic data information for imputation and thus causes the imputation performance loss.

**Results:**

In this study, multiple single imputation methods are combined into an imputation method by ensemble learning. In the ensemble method, the bootstrap sampling is applied for predictions of missing values by each component method, and these predictions are weighted and summed to produce the final prediction. The optimal weights are learned from known gene data in the sense of minimizing a cost function about the imputation error. And the expression of the optimal weights is derived in closed form. Additionally, the performance of the ensemble method is analytically investigated, in terms of the sum of squared regression errors. The proposed method is simulated on several typical genomic datasets and compared with the state-of-the-art imputation methods at different noise levels, sample sizes and data missing rates. Experimental results show that the proposed method achieves the improved imputation performance in terms of the imputation accuracy, robustness and generalization.

**Conclusion:**

The ensemble method possesses the superior imputation performance since it can make use of known data information more efficiently for missing data imputation by integrating diverse imputation methods and learning the integration weights in a data-driven way.

## Background

With the coming of biotechnology era, a lot of gene expression data are generated by DNA microarray technology to measure the expression levels of genes [1]. The analysis of gene expression data has been widely used in numerous researches over a broad range of biological disciplines, including disease diagnosis [2], disease prediction [3], drug design [4], specific therapy identification [5], etc.. However, the available genomics datasets suffer from missing values, which greatly hinder the use of gene data and the mining of effective gene information [6–9].

Genetic data is marked as missing values when the detected signals are very weak or far apart from biological knowledge. That happens due to various factors in the microarray experiment, such as the contamination of microarray surfaces, inappropriate manual operations, insufficient resolution, and systematic errors during the laboratory process, etc. [10–12]. Missing Data recovery is impractical by replicating the microarray experiment because of the high experimental costs and long experimental cycle. Ignoring the rows or columns with missing entries of a matrix of gene data is another optional method in further analysis. However, this results in the significant loss of useful gene information. Thus, as a necessary preprocess operation, missing data imputation is extensively performed before analyzing the microarray data.

So far, many efforts have been made to develop effective imputation methods for missing values in genomics [13–16]. The existing simplest methods are to replace the missing data by zeros, or the average values over the row or column in the target matrix [17]. Obviously, no data structure information is explored in these method. Following the phenomenon that the genes with similar biological function have similar expression profile, the KNNimpute was proposed in [10], which works by computing the weighted average of a set of gene expression data near to those of the target gene. On the basis of KNNimpute, the imputation order for genes with missing data was considered, leading to sequential KNNimpute (SKNNimpute) [18]. Iterative KNNimpute (IKNNimpute) [19] was another variant of KNNimpute, where the predictions of missing data were obtained by iteratively running the KNNimpute method. The later two methods improve the performance of KNNimpute, especially for a large missing rate. Further, by taking dynamic time warping (DTW) distance as the gene matching rule, KNNimpute performs better with respect to the efficiency and accuracy on microarray time-series data [20].

Unlike KNNimpute, a set of neighboring genes were selected by Pearson correlation for a target gene in local least square imputation (LLSimpute) [21], and their relationship was built on a linear regression model. LLSimpute is highly competitive compared to KNNimpute. Moreover, its imputation performance may also be improved by iterative LLSimpute (ILLSimpute) and sequential LLSimpute (SLLSimpute) [18, 22], as done in IKNNimpute and SKNNimpute. Additionally, in [23], the authors presented an imputation framework exploring histone acetylation information, under which performance improvement can be brought about to KNNimpute and LLSimpute.

In [24], missing data imputation was accomplished by integrating decision trees and fuzzy k-means clustering into an iterative learning approach. Comparing with KNNimpute, the method changes the gene matching rule and the imputation model, and achieves the improved accuracy and robustness at the relatively low missing rate.

The above imputation methods have one thing in common, namely, only local similarity structure in gene data set is explored for missing value imputation. On the contrary, some research efforts were made to develop global imputation methods. For example, in singular value decomposition based imputation method (SVDimpute) [10], the missing values of the target genes were represented by a linear combination of a set of mutually orthogonal expression patterns, which are the eigengenes corresponding to the *k* most significant eigenvalues. In comparison with KNNimpute, SVDimpute is relatively sensitive to the missing rate and noise in the data.

In Bayesian principle component analysis (BPCA) [25], a probability model with *k* principal axis vectors was built to model the missing data, and the model parameters were estimated within the framework of Bayesian inference. It has been shown that BPCA outperforms the representative methods mentioned previously. However, the shortcoming of BPCA is that it is difficult to determine the number of principal axes. In [26], the missing data was imputed by applying a global learning with a local similarity measurement module and a global weighted imputation module involved. The method achieves the improved imputation accuracy and less sensitivity to the number of neighbors by contrast with several typical local learning-based imputation methods.

The support vector regression for imputation (SVRimpute) was first developed in [27], where radial basis function was chosen as the kernel function. However, in terms of the prediction accuracy, the method is only comparable with BPCA. SVRimpute was further extended in [28] by modifying the prediction model and the cost function to predict the locations and the values of missing data simultaneously. Relevance vector machine working in the way similar to SVR was also applied for the imputation in [29].

The imputation method based on multilayer perceptron networks (called MLPimpute hereafter) was proposed in [30]. The method learns to establish the mapping from the known data of a gene to its missing data on the whole training dataset. Although multilayer perceptrons have the very good regression performance, the relationship among genes is not considered sufficiently in the method.

A category of hybrid imputation methods was developed by combining local and global learning methods. A typical method is named LinCmb [31], where the final estimates of missing data were produced by integrating the output of five base imputation methods, including row average, KNNimpute, SVDimpute, BPCA and GMCimpute. In [32], the hybrid method works in such a way that the output of BPCA imputation was used to initialize the input of ILLS imputation, thus called BPCA-iLLS. By introducing semi-supervised learning with collaborative training, the recursive mutual imputation (RMI) method was proposed in [33], which exploited the information captured by the two imputation methods used in [32]. The hybrid methods possess the advantages of both local and global learning methods, and thus better adapt to different gene data sets.

There are some works focusing on incorporating the relationships between diverse omics data or biological knowledge for the imputation. In [34], for imputing the missing proteomics data, a Zero-inflated Poisson regression model was built with the use of the correlation between transcriptomics and proteomics datasets. In [35], by a stochastic Gradient Boosted Tree (GBT) method, the relationships between transcriptomics and proteomics data were revealed and used to predict the missing protein abundance. Artificial neural network approach was also applied to impute the missing values of the proteins using the relations between transcriptomics and proteomics data in [36]. Based on ensemble learning, the information from microRNA, mRNA and DNA methylation was combined to estimate the missing data in an integrative model [37]. Obviously, in these methods with more than one gene dataset considered, more information can be explored to improve the imputation performance.

The biological knowledge, such as the functional similarities of genes, the regulatory mechanism, information from multiple external data sets, was applied to the missing data imputation in [38–40]. They help to determine the consistent neighbors or to select top closest genes of a target gene with missing data. However, such kind of imputation methods requires domain-specific knowledge and are infeasible for the situations without or less prior knowledge.

Notice that most of the existing imputation methods make use of only a certain characteristic of the genetic data to impute the missing values, resulting in the weak generalization or even the database-dependent performance. To solve the problem, a comprehensive method based on ensemble learning is proposed in this paper. First, a set of representative single imputation methods are built and individually applied for predicting the missing values with the use of the bootstrap sampling. Then, the predictions output by all the individual predictors are combined into the final prediction using weighted average. And the weights for the linear prediction model are learned by using this model to estimate known gene data and minimizing the imputation errors. The proposed method has two prominent advantages: (1) more information from known genomics data is allowed to be used for the performance improvement; (2) the good generalization can be achieved by a weight learning approach involved in the training procedure.

The main contributions of this work are as follows:

1. A basic framework for the ensemble learning based imputation method is proposed, where bootstrap sampling is introduced to train a set of base predictors, and the base predictors are integrated by the weighted average. On the framework, a strong predictor can be derived by the combinations of weak base predictors.
2. The learning scheme of the combination weights is provided for the ensemble imputation. In this scheme, a linear regression model is built for the combination weights, and the expression of the optimal weights is derived in closed form.
3. A specific ensemble imputation method is carefully described, including the choice of base predictors and the generation of multiple implementations for each predictor. The proposed method is extensively tested on several typical genomic datasets, and compared with the state-of-the-art imputation methods. The experiments confirms that our method achieves the significant performance improvement.

The remainder of this paper is structured as follows. First, the problem model for missing value imputation is given and some basic definitions and conventions are formulated. Next, the ensemble imputation method with the bootstrap sampling is presented. Here, the imputation procedure and the weight learning scheme are carefully described. Detailed derivation of the optimal weights is provided in the sequel. The theoretical performance is subsequently analyzed in terms of the imputation errors. In addition, the choice of base imputation methods and the generation of the base predictions are explained. After that, a series of tests are done to evaluate the presented method. Finally, we conclude the paper.

## Problem model

Throughout the article, we will use a matrix ${\pmb {G}}\in {\mathcal {R}}^{M\times N}$ to denote the gene expression data for *M* genes in *N* experiments. The element of ${\pmb {G}}$ at the position (*i*, *j*) is designated by $g_{i,j}$, which is the data for the *i*th gene produced in the *j*th experiment.

Due to various reasons, e.g., media or experimental conditions, the elements of ${\pmb {G}}$ are not completely known. The missing values of the *i*th gene locate at the *i*th row of ${\pmb {G}}$ and columns whose positions compose the set $\Omega _i$. The complementary set of $\Omega _i$, denoted by ${\bar{\Omega }}_i$, contains the column positions of the known values of the *i*th gene. The missing rate $\gamma$ is thus expressed as $\gamma =\frac{1}{MN}\sum _{i=1}^M|\Omega _i|$, where $|\Omega _i|$ represents the cardinality of $\Omega _i$.

Further, for the sake of explanation, a vector or matrix operator $(\cdot )_\cdot$ represented by ${\pmb {y}}=({\pmb {x}})_{\varphi }$ or ${\pmb {Y}}=({\pmb {X}})_{\varphi }$ is introduced, which means that the vector ${\pmb {y}}$ (or matrix ${\pmb {Y}}$) is produced by extracting the elements (or columns) of a given vector ${\pmb {x}}$ (matrix ${\pmb {X}}$) at the positions in the set $\varphi$. By the operator, the vectors ${\pmb {g}}_i$ and ${\pmb {g}}_i$, which are respectively composing of the missing values and the known values of gene *i*, can be written as ${\pmb {g}}_i=\left( g_{i,1},g_{i,2},\ldots ,g_{i,N}\right) _{\Omega _i}$ and $\widetilde{{\pmb {g}}}_i=\left( g_{i,1},g_{i,2},\ldots ,g_{i,N}\right) _{{\bar{\Omega }}_i}$. The vector ${\pmb {g}}=({\pmb {g}}_1,{\pmb {g}}_2,\ldots ,{\pmb {g}}_M)$ is thus composed of all missing elements in ${\pmb {G}}$.

The basic idea of missing value imputation is to estimate the missing gene expression data by the use of the known gene expression data. Using the above notations, the process can be generally expressed as

1 $$\begin{aligned} \hat{{\pmb {g}}}={\mathcal {H}}(\widetilde{{\pmb {g}}}_1,\widetilde{{\pmb {g}}}_2,\ldots ,\widetilde{{\pmb {g}}}_M), \end{aligned}$$

where $\hat{{\pmb {g}}}$ denotes the imputation vector for ${\pmb {g}}$. The imputation function ${\mathcal {H}}(\cdot )$ is usually built by minimizing a certain cost function of $\widetilde{{\pmb {g}}}_i$, $i=1,2,\ldots ,M$.

The performance of an imputation method is usually assessed by the normalized root mean square error (NRMSE), which is the most widely used metric to evaluate the accuracy of a prediction approach. For the imputation problem, NRMSE is defined as

2 $$\begin{aligned} \text {NRMSE} =\frac{\Vert \hat{{\pmb {g}}}-{\pmb {g}}\Vert }{\sqrt{MN\gamma \text {Var}\left( {\pmb {g}} \right) }}, \end{aligned}$$

where $\Vert \cdot \Vert$ stands for Euclidean (i.e., $\ell _2$) norm, and $\text {Var}(\cdot )$ is the sample variance operator. Obviously, NRMSE can reflect the estimation accuracy by the root mean square errors between the imputation values and the true values, and the impact from the dispersion degree of the true gene expression data.

## Methods

### Ensemble imputation

**Fig. 1:**
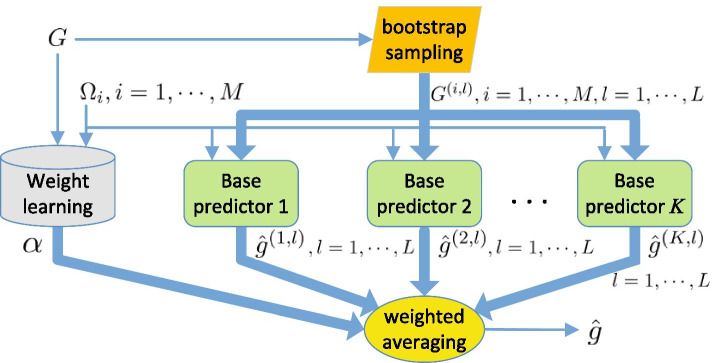
Basic framework of ensemble imputation

As a major learning paradigm, an ensemble method tries to construct a set of learners from training data and combine them to generate a desirable learner [41]. The prominent advantage of ensemble methods is that weak learners can be boosted to a strong learner [41, 42]. Following the same idea, we develop an ensemble method for missing value imputations. The whole imputation process is shown in Fig. 1, and carefully described as follows.

Step 1: A set of *K* imputation methods are selected as the component predictors in the proposed ensemble method. According to the generalization error analysis for ensemble learning in [42], the use of independent component predictors can dramatically reduce the prediction errors. The selected component predictors will be described in a later section.

Step 2: In order to predict ${\pmb {g}}_i$ of gene *i* by each component predictor multiple times, *L* samples ${\pmb {G}}^{(i,l)}$, $l=1, 2, \ldots , L$ of the given gene express data in ${\pmb {G}}$ are generated in such a way that ${\pmb {G}}^{(i,l)}=\left( {\pmb {G}}\right) _{\Omega _i\cup {\bar{\Omega }}_i^{(l)}}$, where ${\bar{\Omega }}_i^{(l)}$ is the *l*th sampled set of the known column position set ${\bar{\Omega }}_i$. Here, the bootstrap sampling is adopted for the generation of ${\bar{\Omega }}_i^{(l)}$. In such a sampling way, randomness can be introduced into the process for building the component predictors, which is in favor of the reduction of their dependence.

Step 3: For the *k*th imputation method, the imputation function $h^{(k,l)}_i$ is built for gene *i* with the use of the data in sample ${\pmb {G}}^{(i,l)}$. The detailed explanations will be presented in each individual base method. Therefore, the estimation $\hat{{\pmb {g}}}^{(k,l)}_i$ of the missing vector ${\pmb {g}}_i$ is obtained by applying

3 $$\begin{aligned} \hat{{\pmb {g}}}^{(k,l)}_{i}=h^{(k,l)}_i\left( {\pmb {G}}^{(i,l)}\right) . \end{aligned}$$

Step 4: By weighting and summing the predictions in (3), the final prediction $\hat{{\pmb {g}}}$ of ${\pmb {g}}$ is produced as

4 $$\begin{aligned} \hat{{\pmb {g}}}={\pmb {\alpha }}\hat{{\pmb {G}}}, \end{aligned}$$

where ${\pmb {\alpha }}$ denotes the row weight vector of length $K\times L$, and $\hat{{\pmb {G}}}$ is a matrix with the $\left( (k-1)*L+l\right)$th row being the vector $\hat{{\pmb {g}}}^{(k,l)}=(\hat{{\pmb {g}}}_1^{(k,l)}, \hat{{\pmb {g}}}_2^{(k,l)}, \ldots , \hat{{\pmb {g}}}_M^{(k,l)})$. A large weight component means that the corresponding imputation method has a high priority. To obtain an optimal weight vector is of crucial significance for the ensemble method, and will be presented in the following section.

A few observations are in order about the proposed imputation method. First, in step 2, the sample datasets are generated by bootstrap sampling for each utilized base imputation method, by which the performance loss of them from sampling process can be reduced.

Second, the predictions given by the individual predictors are combined into the final prediction in step 4. It has been theoretically shown [41] that the variance and the bias of the final prediction errors can be reduced by the integration.

A more intuitive explanation of the theoretical results is that each individual predictor is only adapted to a data space with a certain characteristics and the combination of them is capable of expressing a data space with various characteristics and forming a better imputation method. The specific performance analysis for the ensemble method will be addressed later.

Third, the optimal weight vector ${\pmb {\alpha }}$ is obtained by a learning approach on a given data matrix, and thus takes different values on different datasets. As a result, a better generalization ability can be achieved by the ensemble method [42].

In addition, Equation (4) indicates that a set of *L* predictions obtained by a base imputation method are combined with the use of different weights, while the same weight is assigned to the predictions of a base learner in the existing stacked regression methods. From this perspective, the proposed imputation method utilizes a more general combination rule.

### Weight learning for base imputation methods

In the expression (4), the weight vector ${\pmb {\alpha }}$ is unknown and should be learned from known gene express data in the dataset ${\pmb {G}}$. To be specific, a set of known gene data of the matrix ${\pmb {G}}$ are randomly chosen to form a vector ${\varvec{{g}}}$ as we construct the missing vector ${\pmb {g}}$. First, by applying $h_i^{(k,l)}(\cdot )$, $i=1,\ldots ,M$, the prediction $\hat{{\varvec{{g}}}}^{(k,l)}=(\hat{{\varvec{{g}}}}_1^{(k,l)}, \ldots , \hat{{\varvec{{g}}}}_M^{(k,l)})$ of ${\varvec{{g}}}$ is generated. In our simulations, the prediction of all the known data are taken to derive the good combination weights. That is, the vector $\hat{{\varvec{{g}}}}^{(k,l)}$ is composed of the predictions of all the known data. Then, similarly to $\hat{{\pmb {G}}}$, the matrix $\hat{{\pmb {G}}}_T$ is formed by the use of $\hat{{\varvec{{g}}}}^{(k,l)}$, $k=1,2,\ldots ,K$, $l=1,2,\ldots , L$. Last, the weight vector ${\pmb {\alpha }}$ is determined in order to minimize the imputation error as

5 $$\begin{aligned} {\pmb {\alpha }}=\arg \min \limits _{{\pmb {\alpha }}'}\Vert {\varvec{{g}}}-{\pmb {\alpha }}'\hat{{\pmb {G}}}_T\Vert ^2 \end{aligned}$$

subject to the conditions

6 $$\begin{aligned} \forall \, i, \alpha _{i}\ge 0 \,\text {and}\, \sum \limits _{i=1}^{KL}\alpha _{i}=1, \end{aligned}$$

where $\alpha _{i}$ is the *i*th element of ${\pmb {\alpha }}$.

This is a convex optimization problem with linear constraints. Solving the problem yields

7 $$\begin{aligned} {\pmb {\alpha }}^T={\pmb {\alpha }}_0^T+{\pmb{B}}{\pmb{\lambda }}^T \end{aligned}$$

where

$$\begin{aligned} {\pmb {\alpha }}_0^T={\pmb{A}}^{\dagger }\hat{{\pmb {G}}}_T{\varvec{{g}}}^T-\frac{{\pmb{1}}{\pmb{A}}^{\dagger }\hat{{\pmb {G}}}_T{\varvec{{g}}}^T-1}{{\pmb{1}}{\pmb{A}}^{\dagger }{\pmb{1}}^T}{\pmb{A}}^{\dagger }{\pmb{1}}^T \end{aligned}$$

and

$$\begin{aligned} {\pmb{B}}={\pmb{A}}^{\dagger }-\frac{{\pmb{A}}^{\dagger }{\pmb{1}}^T{\pmb{1}}{\pmb{A}}^{\dagger }}{{\pmb{1}}{\pmb{A}}^{\dagger }{\pmb{1}}^T} \end{aligned}$$

with ${\pmb{1}}=(1,1,\ldots ,1)$, ${\pmb{A}}=\hat{{\pmb {G}}}_T\hat{{\pmb {G}}}_T^T$ and the superscript $\dagger$ denoting the pseudo inverse operator.

In (7), the vector ${\pmb{\lambda }}$ has zero components located at the columns designated by the elements in the set ${\overline{\Psi }}$, and other non-zero elements determined according to

8 $$\begin{aligned} \left( ({\pmb{\lambda }})_{\Psi }\right) ^T=-\left( \left[ ({\pmb{B}})_{\Psi }\right] _{\Psi }\right) ^{\dagger }\left( ({\pmb {\alpha }}_0)_{\Psi }\right) ^T \end{aligned}$$

where the sets $\Psi$ and ${\overline{\Psi }}$ satisfy $\left( {\pmb{B}}^{\dagger }{\pmb {\alpha }}_0^T\right) _i<0, \forall \, i\in \Psi$, and ${\overline{\Psi }}={\mathcal {C}}_M-\Psi$ with ${\mathcal {C}}_M$ being the complete set, defined as ${\mathcal {C}}_M=\{1,2,\ldots ,KL\}$. In the formula (8), the term $\left[ ({\pmb{B}})_{\Psi }\right] _{\Psi }$ represents the matrix formed by the rows of the matrix $({\pmb{B}})_{\Psi }$ listed in the set $\Psi$.

The detailed derivation is presented as follows. To solve the optimization problem (5) with the constraints in (6), we build the associated Lagrangian ${\mathcal {L}}(\cdot )$ as

9 $$\begin{aligned} {\mathcal {L}}({\pmb {\alpha }}',\eta ,{\pmb{\lambda }})=\frac{1}{2}\Vert {\varvec{{g}}}-{\pmb {\alpha }}'\hat{{\pmb {G}}}_T\Vert ^2+\eta ({\pmb {\alpha }}'{\pmb{1}}^T-1)-{\pmb {\alpha }}'{\pmb{\lambda }}^T, \end{aligned}$$

where $\eta$ refers to the Lagrange multiplier associated with the equality constraint in (6), and ${\pmb{\lambda }}$ is a Lagrange multiplier vector associated with the inequality constraint in (6). The Karush–Kuhn–Tucker (KKT) conditions state that the optimal points for ${\pmb {\alpha }}'$, $\eta$ and ${\pmb{\lambda }}$ must satisfy

10 $$\frac{\partial {\mathcal {L}}({\pmb {\alpha }}',\eta ,{\pmb{\lambda }})}{\partial {\pmb {\alpha }}'}= 0 $$

11 $$\lambda_i  \ge0,\,\,i=1,\ldots ,K$$

12 $$ \lambda _i\alpha _i \ge 0,\,\, i=1,\ldots ,K $$

where $\alpha _i'$ and $\lambda _i$ denote the *i*th component of the vector ${\pmb {\alpha }}'$ and ${\pmb{\lambda }}$, respectively.

According to (9), we can write

$$\begin{aligned} \frac{\partial {\mathcal {L}}({\pmb {\alpha }}',\eta ,{\pmb{\lambda }})}{\partial {\pmb {\alpha }}'}=\left( {\pmb {\alpha }}'\hat{{\pmb {G}}}_T-{\varvec{{g}}}\right) \hat{{\pmb {G}}}_T^T+\eta {\pmb{1}}-{\pmb{\lambda }} \end{aligned}$$

With the constraint (10), it is easy to derive

13 $$\begin{aligned} {\pmb {\alpha }}'=\left( {\varvec{{g}}}\hat{{\pmb {G}}}_T^T-\eta {\pmb{1}}+{\pmb{\lambda }}\right) \left( {\pmb{A}}^{\dagger }\right) ^T \end{aligned}$$

Combining the equality constraints in (6) and (13), we have

14 $$\begin{aligned} \eta =\frac{{\pmb{1}}{\pmb{A}}^{\dagger }\hat{{\pmb {G}}}_T{\varvec{{g}}}^T+{\pmb{1}}{\pmb{A}}^{\dagger }{\pmb{\lambda }}^T-1}{{\pmb{1}}{\pmb{A}}^{\dagger }{\pmb{1}}^T} \end{aligned}$$

Substituting (14) into (13), results in (7).

Further, applying the inequality in (6) to (7), we obtain

$$\begin{aligned} {\pmb{\lambda }}^T\ge -{\pmb{B}}^{\dagger }{\pmb {\alpha }}_0^T \end{aligned}$$

By considering the inequality (11) and due to (12), it is immediate to write $\lambda _i=0$ if $\left( {\pmb{B}}^{\dagger }{\pmb {\alpha }}_0^T\right) _i\ge 0$ and $\alpha _i=0$ otherwise, $i=1,2,\ldots ,K$. That is,

15 $$\begin{aligned} \begin{array}{rcl} ({\pmb{\lambda }})_{{\overline{\Psi }}}&{}=&{}(0,0,\ldots ,0)\\ ({\pmb {\alpha }})_{\Psi }&{}=&{}(0,0,\ldots ,0) \end{array} \end{aligned}$$

Last, (8) can be obtained by inserting (7) into (15) and solving the equation.

### Theoretical analysis for imputation error

In the sequel, the theoretical performance for the ensemble imputation method is accessed by the sum of squared regression errors, denoted by $E_r$. According to (3) and (4), we can write

16 $$\begin{aligned} E_r=E\left\{ \sum \limits _{i=1}^M\left\| \sum \limits _{j=1}^{KL}\alpha _{j}\hat{{\pmb {g}}}^{(k,l)}_{i}-{\pmb {g}}_i\right\| ^2\right\} , \end{aligned}$$

where $E\{\cdot \}$ is the expectation operator, and the parameters *k* and *l* satisfy $j=(k-1)L+l$. For an individual predictor $h^{(k,l)}$, the sum of squared regression errors $E_{r}^{(k,l)}$ becomes

17 $$\begin{aligned} E_{r}^{(k,l)}=E\left\{ \sum \limits _{i=1}^M\left\| \hat{{\pmb {g}}}^{(k,l)}_{i}-{\pmb {g}}_i\right\| ^2\right\} . \end{aligned}$$

Comparing (16) to (17), it is easy to derive

18 $$\begin{aligned} \min \limits _{\mathbf {\alpha }}E_r\le \min \limits _{k,l}E_{r}^{(k,l)}. \end{aligned}$$

The expression (18) shows that the ensemble method in statistics can perform better than the strongest individual predictor among the used predictors by choosing the optimal combination weights.

Further, let $\bar{{\pmb {g}}}_{i}=\sum \nolimits _{j=1}^{KL}\alpha _{j}E\left\{ \hat{{\pmb {g}}}^{(k,l)}_{i}\right\}$ and $\hat{{\pmb {g}}}_{i}=\sum \nolimits _{j=1}^{KL}\alpha _{j}\hat{{\pmb {g}}}^{(k,l)}_{i}$. Then, the equivalent expression for $E_r$ in (16) is

19 $$\begin{aligned} E_r& {} = E\left\{ \sum \limits _{i=1}^M\left\| \hat{{\pmb {g}}}_{i}-\bar{{\pmb {g}}}_{i}+\bar{{\pmb {g}}}_{i}-{\pmb {g}}_i\right\| ^2\right\} \\& {} = \sum \limits _{i=1}^ME\left\{ \left\| \hat{{\pmb {g}}}_{i}-\bar{{\pmb {g}}}_{i}\right\| ^2\right\} +2E\left\{ \left( \hat{{\pmb {g}}}_{i}-\bar{{\pmb {g}}}_{i}\right) \right\} \left( \bar{{\pmb {g}}}_{i}-{\pmb {g}}_i\right) ^T \\&\quad +E\left\{ \left\| \bar{{\pmb {g}}}_{i}-{\pmb {g}}_i\right\| ^2\right\} \\& {} = \sum \limits _{i=1}^ME\left\{ \left\| \hat{{\pmb {g}}}_{i}-\bar{{\pmb {g}}}_{i}\right\| ^2\right\} +\sum \limits _{i=1}^ME\left\{ \left\| \bar{{\pmb {g}}}_{i}-{\pmb {g}}_i\right\| ^2\right\} . \end{aligned}$$

Moreover, it is easy to derive that

20 $$\begin{aligned} \sum \limits _{i=1}^ME\left\{ \left\| \hat{{\pmb {g}}}_{i}-\bar{{\pmb {g}}}_{i}\right\| ^2\right\} =E_{r_1}+E_{r_2}, \end{aligned}$$

where

$$\begin{aligned} E_{r_1}=\sum \limits _{i=1}^M\sum \limits _{j=1}^{KL}\alpha _{j}^2E\left\{ \left\| \hat{{\pmb {g}}}^{(k,l)}_{i}-E\left\{ \hat{{\pmb {g}}}^{(k,l)}_{i}\right\} \right\| ^2\right\} \end{aligned}$$

and

$$\begin{aligned} E_{r_2}& {} = \sum \limits _{i=1}^M\sum \limits _{j=1}^{KL}\sum \limits _{\begin{array}{c} j'=1\\ j'\ne j\end{array}}^{KL}\alpha _{j}\alpha _{m}E\left\{ \left( \hat{{\pmb {g}}}^{(k,l)}_{i}-E\left\{ \hat{{\pmb {g}}}^{(k,l)}_{i}\right\} \right) \right. \\\times & {} \left. \left( \hat{{\pmb {g}}}^{(k',l')}_{i}-E\left\{ \hat{{\pmb {g}}}^{(k',l')}_{i}\right\} \right) ^T\right\} \end{aligned}$$

with the parameters $k'$ and $l'$ satisfying $j'=(k'-1)L+l'$. As a result, $E_r$ can be decomposed into three terms as

21 $$\begin{aligned} E_r=E_{r_1}+E_{r_2}+E_{r_3}, \end{aligned}$$

where

$$\begin{aligned} E_{r_3}=\sum \limits _{i=1}^ME\left\{ \left\| \sum \limits _{j=1}^{KL}\alpha _{j}\left( E\left\{ \hat{{\pmb {g}}}^{(k,l)}_{i}\right\} -{\pmb {g}}_i\right) \right\| ^2\right\} . \end{aligned}$$

The three terms $E_{r_1}$, $E_{r_2}$ and $E_{r_3}$ in (21) can be called the variance, covariance and bias terms, respectively, according to their expressions. Apparently, by choosing a set of strong base predictors, the variance term $E_{r_1}$ and the bias term $E_{r_3}$ can be effectively reduced. The covariance term $E_{r_2}$ actually models the correlation between the chosen base predictors, and the base predictors making different errors are preferred. The diversity is obtained by applying the bootstrap sampling to the generation of the training samples as well as choosing the relatively independent base predictors in our ensemble method. Through the above analyses, it can be understood that the proposed ensemble method has the significant performance advantage over an individual predictor.

In addition, we may assess the effectiveness of the ensemble method by the estimation bias $\epsilon$, which is calculated by

22 $$\begin{aligned} \epsilon& {} = \frac{1}{M}\sum \limits _{i=1}^ME\left\{ \sum \limits _{j=1}^{KL}\alpha _{j}\hat{{\pmb {g}}}^{(k,l)}_{i}-{\pmb {g}}_i\right\} \\& {} = \frac{1}{M}\sum \limits _{i=1}^M\sum \limits _{j=1}^{KL}\alpha _{j}\left( E\left\{ \hat{{\pmb {g}}}^{(k,l)}_{i}\right\} -{\pmb {g}}_i\right) . \end{aligned}$$

The expression (22) shows that the bias for the proposed method is the weighted average of the biases for the utilized base predictors. Therefore, if each base predictor is unbiased, the output of the proposed method is also unbiased. And the bias in estimation can be reduced by choosing the base predictors with small biases.

### Utilized individual imputation methods

To design the ensemble method, four early and relatively primitive imputation methods are adopted as base predictors: KNN imputation [10], LLS imputation [21], ILLS imputation [22] and SVD imputation [10]. They were developed following the relatively independent ideas and work independently to some extent. The first three methods explore the *P* nearest neighbor genes, i.e., the local gene information, for the imputation, however, they determine the candidate genes by different gene matching rules. The last one achieves the aim by the use of the support vectors corresponding to the *Q* largest singular values, which contains the global gene information. As a result, the key characteristic diversity of base predictors can be ensured to obtain a good ensemble. Detailed descriptions for the chosen base predictors are presented as follows.

#### KNNimpute

We establish the imputation functions $h_i^{(1,l)}(\cdot )$ on the dataset ${\pmb {G}}^{(i,l)}$, $l=1,2,\ldots , L$ by KNNimpute. First, the missing values of ${\pmb {G}}^{(i,l)}$ except for those of gene *i* should be filled for the neighboring gene searching. As in [10], the row average approach is adopted.

Then, taking the vector $\widetilde{{\pmb {g}}}^{(l)}_{i}$ with the elements of the matrix at the *i*th row and the columns in ${\bar{\Omega }}_i$ after the row average operation, the Euclidean distance between gene *i* and each of other genes is computed as $d_{ij}^{(l)}= \Vert \widetilde{{\pmb {g}}}^{(l)}_{i}-\widetilde{{\pmb {g}}}^{(l)}_{j}\Vert$, $j=1,\ldots ,M$ and $j\ne i$, where the vector $\widetilde{{\pmb {g}}}^{(l)}_{j}$ is defined similarly to $\widetilde{{\pmb {g}}}^{(l)}_{i}$.

Next, by Euclidean distance, the *P* nearest neighbor genes of gene *i* are determined as the candidate genes for the imputation, whose expression data composes a matrix of size $P\times N$, denoted by ${\pmb {G}}^{(i,l)}_{c}$.

Last, the missing gene data for the *i*th gene are estimated by

23 $$\begin{aligned} \hat{{\pmb {g}}}^{(1,l)}_{i}={\pmb {\beta }}^{(1,l)}_i\left( {\pmb {G}}^{(i,l)}_{c}\right) _{\Omega _i}, \end{aligned}$$

where ${\pmb {\beta }}^{(1,l)}_i$ is a row vector of weights corresponding to the *i*th gene and the *l*th sampling. For KNNimpute, the *j*th element of the weight vector ${\pmb {\beta }}^{(1,l)}_i$ is given by $1/d_{i,j}^{(l)}/\sum _{j=1}^P1/d_{i,j}^{(l)}$ with $d_{i,j}^{(l)}$ being the distance between $\widetilde{{\pmb {g}}}^{(l)}_{i}$ and the *j*th row of ${\pmb {G}}^{(i,l)}_{c}$.

Since KNNimpute estimates the missing data by exploiting the local structure information in the target dataset, the imputation performance largely depends on the local similarity of gene data. Moreover, it is clearly unable to make use of the global information contained in the data.

#### LLSimpute

We use LLSimpute to establish the imputation functions $h_i^{(2,l)}(\cdot )$ on the sample dataset ${\pmb {G}}^{(i,l)}$, $l=1,2,\ldots , L$. The basic imputation process is similar to that of KNNimpute but the utilized gene matching rule and the computation of the weight vector.

Specifically, in LLSimpute, Pearson correlation based gene matching rule is adopted to find out the *P* nearest neighbor genes of gene *i*. For any two genes *i* and *j*, the Pearson correlation $\delta _{ij}^{(l)}$ is obtained by computing the inner product between the normalized versions of $\widetilde{{\pmb {g}}}^{(l)}_{i}$ and $\widetilde{{\pmb {g}}}^{(l)}_{j}$.

The weight vector ${\pmb {\beta }}^{(2,l)}_i$ is derived so that

24 $$\begin{aligned} {\pmb {\beta }}^{(2,l)}_i=\arg \min \limits _{{\pmb {\beta }}}\Vert \widetilde{{\pmb {g}}}^{(l)}_{i}-{\pmb {\beta }}{\pmb {G}}^{(i,l)}_{c}\Vert ^2, \end{aligned}$$

where ${\pmb {G}}^{(i,l)}_{c}$ is the candidate gene data matrix determined by the Pearson correlation based gene matching rule. Solving (24) yields ${\pmb {\beta }}^{(2,l)}_i=\widetilde{{\pmb {g}}}^{(l)}_{i}\left( {\pmb {G}}^{(i,l)}_{c}\right) ^{\dagger }$.

Clearly, LLSimpute is a local imputation method with the use of the correlation among gene data. It has the same shortcomings as KNNimpute, and is more sensitive to the number of reference genes and the missing rate.

#### ILLSimpute

The imputation function $h_i^{(3,l)}(\cdot )$ is built on the sample dataset ${\pmb {G}}^{(i,l)}$, $l=1,2,\ldots , L$ by ILLSimpute. ILLSimpute is an iterative missing value imputation method. At each iteration, ILLSimpute updates the candidate gene dataset ${\pmb {G}}^{(i,l)}_{c}$ by applying Pearson correlation based gene matching rule to the imputed matrix at previous iteration. Then, it is substituted into (23) and (24) to derive the new imputation results. This procedure is carried out iteratively until a pre-defined quantity of iterations is reached or there are no differences of imputed values between two iterations [22].

It has been shown that ILLSimpute achieves the improved imputation quality by multiple iterations of imputation, but fails to capture some unique properties that non time series datasets have. That is, ILLSimpute presents the good performance only on some kind of datasets.

#### SVDimpute

SVDimpute is finally used to construct the imputation functions $h_i^{(4,l)}(\cdot )$ on the sample dataset ${\pmb {G}}^{(i,l)}$, $l=1,2,\ldots , L$. The first step is to fill the missing values of ${\pmb {G}}^{(i,l)}$, resulting in ${\pmb {G}}^{(i,l)}_{r'}$. Unlike KNNimpute, all the missing values of ${\pmb {G}}^{(i,l)}$ are filled for singular value decomposition (SVD).

Then, the resulted real matrix ${\pmb {G}}^{(i,l)}_{r'}$ is decomposed by applying SVD , expressed as

25 $$\begin{aligned} {\pmb {G}}^{(i,l)}_{r'}={\pmb{U}}^{(i,l)} \Sigma ^{(i,l)} {{\pmb{V}}^{(i,l)}}^T, \end{aligned}$$

where ${\pmb{U}}^{(i,l)}$ and ${\pmb{V}}^{(i,l)}$ are orthogonal matrices of size $M\times M$ and $N\times N$ respectively, and ${\pmb{\Sigma}}^{(i,l)}$ is a diagonal matrix of size $\min \{M,N\}\times \min \{M,N\}$. The diagonal elements of ${\pmb{\Sigma}}^{(i,l)}$ consist of non-negative singular values of ${\pmb {G}}^{(i,l)}_{r'}$.

Next, the eigengenes corresponding to the *Q* largest eigenvalues are selected from ${{\pmb{V}}^{(i,l)}}^T$ to construct the matrix ${{\pmb{V}}_c^{(i,l)}}^T$. And the prediction $\hat{{\pmb {g}}}^{(4,l)}_{i}$ for the missing gene data ${\pmb {g}}_i$ of gene *i* can be represented by (23), where ${\pmb {G}}^{(i,l)}_{c}$ is replaced by ${{\pmb{V}}_c^{(i,l)}}^T$, and the weight vector is denoted by ${\pmb {\beta }}_i^{(4,l)}$.

Last, the weight vector ${\pmb {\beta }}_i^{(4,l)}$ is optimized as done in LLSimpute. Thus, ${\pmb {\beta }}_i^{(4,l)}$ is expressed by replacing ${\pmb {G}}^{(i,l)}_{c}$ in the expression of ${\pmb {\beta }}_i^{(2,l)}$ with ${{\pmb{V}}_c^{(i,l)}}^T$.

SVDimpute is suitable for a large microarray dataset having a good global structure. It is relatively sensitive to noise in the data. Moreover, it often manifests unsatisfactory performance on the dataset with similar local structures.

Now, we can see the distinct difference between the proposed ensemble method and the typical hybrid imputation method, LinCmb [31]. LinCmb predicts the missing data by a different combination of five imputation methods, and three of them are not applied in our method. Moreover, in the process, bootstrap sampling is not explored, and thus it is difficult to ensure that the diversity of base predictors and the randomness of the prediction errors are obtained. Meanwhile, the expression for the optimal weights is not derived in closed form in LinCmb.

## Results

### Simulation scheme

Simulations are carried out on a complete data matrix containing 50 subjects with 104 microRNAs (miRNA). The data matrix is derived by the use of a subset of the cancer genomic atlas database on Glioma cancer study with all missing values removed [43], called TCGA subset1. The incomplete data matrix is generated from the complete one by randomly removing a set of entries at a certain missing rate. And the proposed ensemble method is applied to impute the faking missing data. The performance is measured by the average NRMSE over 30 imputations. We investigate the imputation performance by varying the missing rate and the sample size, as well as adding Gaussian noise with different standard deviations to the incomplete data matrix. The results of KNNimpute [10], LLSimpute [21], ILLSimpute [22], and SVDimpute [10] are also presented for comparison purposes.

### Parameter setting

The bootstrap sampling is performed $T=30$ times for computing the average weight vector ${\pmb {\alpha }}$ except otherwise indicated. Note that better imputation performance can be achieved by increasing *T* but the larger computational cost will be caused. The parameters for the utilized component imputation methods KNNimpute and SVDimpute take the optimal values as suggested in [10]. That is, the neighboring size $P=15$ is taken for KNNimpute, and the number *Q* of the selected eigengenes is $20\%$ of the samples number in SVDimpute. For LLSimpute and ILLSimpute, we simply set the same neighboring size *P* as that of KNNimpute for avoiding the optimal parameter searching as done in [21, 22]. The number of iterations for ILLSimpute is set to 10. These parameter settings remain the same when each base imputation method is individually applied for imputation.

### Performance evaluation

**Fig. 2:**
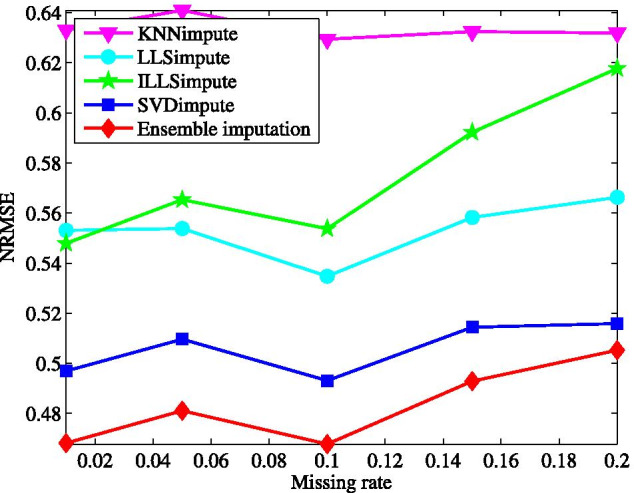
Average NRMSE by KNNimpute [10], LLSimpute [21], ILLSimpute [22], SVDimpute [10], and ensemble imputation on TCGA subset1 with different missing rates

First, we test the imputation performance by varying the missing rate from 1 to 20%. The results for all the tested methods are shown in Fig. 2. Clearly, the ensemble method yields the best performance on the microRNAs data matrix among all the tested methods. In the wide range of the missing rate, the ensemble method presents the lowest NRMSE. This performance advantage is brought about by combining the individual imputation methods in the ensemble learning way. With the increase of missing rate, the performance of all the methods becomes worse, particularly for ILLSimpute. This is caused by the fact that less data information can be explored for imputation at a larger missing rate. In contrast, the performance of our method degrades more gradually as the missing rate increases.

**Fig. 3:**
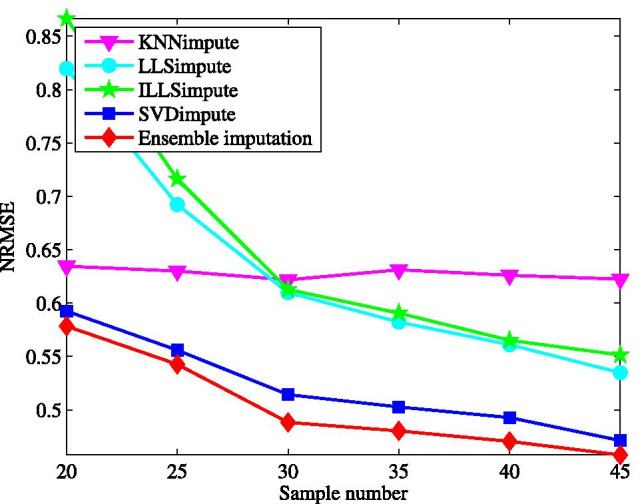
Average NRMSE by KNNimpute [10], LLSimpute [21], ILLSimpute [22], SVDimpute [10], and ensemble imputation on TCGA subset1 with different sample number

Next, the imputation performance is evaluated for different sample size, where the incomplete data matrix is generated at a given missing rate after a specific number of samples are selected randomly. The effect of sample size varying from 20 to 45 is demonstrated in Fig. 3 with the missing rate at $5\%$. We can see, among the tested methods, ILLSimpute seems to be the most sensitive to sample size, particular for sample size lower than 30. It is easy to be understood that a small sample size may lead to over-fitting for local methods, like LLSimpute, while for SVDimpute the incomplete data matrices tends to be ill-conditioned in the case of less samples. Although the NRMSE for the proposed method increases as sample size decreases, it performs better than other individual methods across all different sample size.

**Fig. 4:**
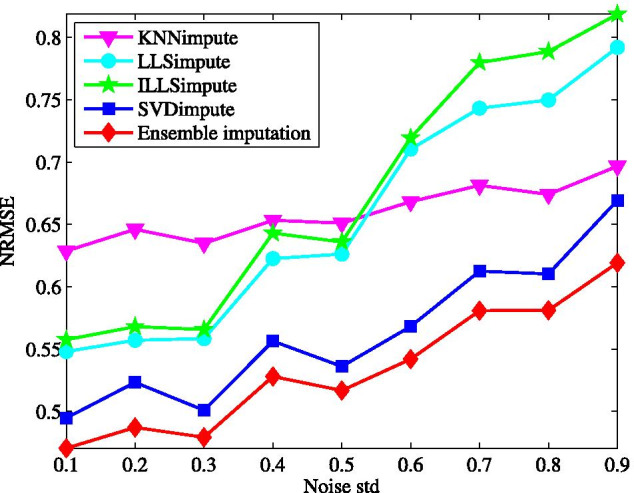
Average NRMSE by KNNimpute [10], LLSimpute [21], ILLSimpute [22], SVDimpute [10], and ensemble imputation on TCGA subset1 with different noise of standard deviation

**Fig. 5:**
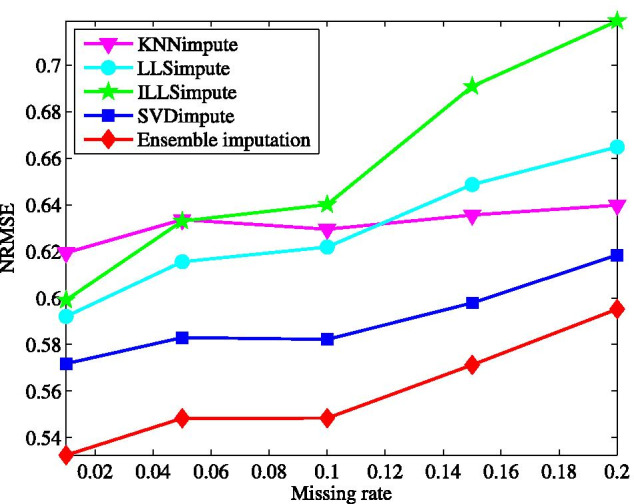
Average NRMSE by KNNimpute [10], LLSimpute [21], ILLSimpute [22], SVDimpute [10], and ensemble imputation on TCGA subset2 with different missing rates

**Fig. 6:**
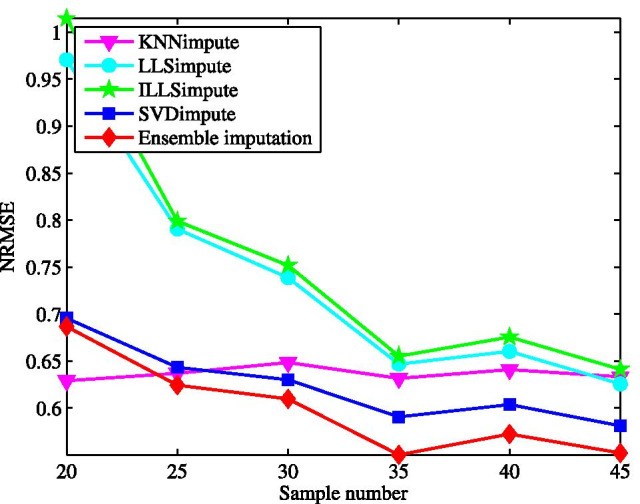
Average NRMSE by KNNimpute [10], LLSimpute [21], ILLSimpute [22], SVDimpute [10], and ensemble imputation on TCGA subset2 with different sample number

**Fig. 7:**
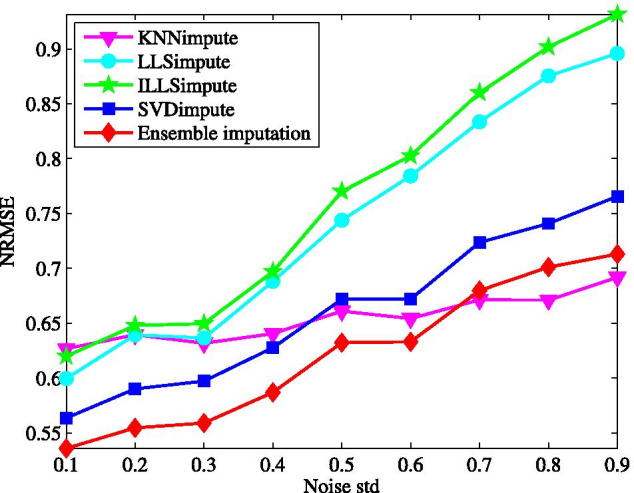
Average NRMSE by KNNimpute [10], LLSimpute [21], ILLSimpute [22], SVDimpute [10], and ensemble imputation on TCGA subset2 with different noise of standard deviation

Last, considering the measurement of gene expression data itself has a lot of noises, the imputation performance shall be evaluated in the existence of noise. For this purpose, the generated incomplete data matrices undergo additive white Gaussian noise (AWGN) with standard deviation being within the range 0.1–0.9. The robustness of all the tested methods to noise is demonstrated in Fig. 4 while fixing the missing rate at $5\%$. Clearly, the stronger the noise is, the worse the ensemble method performs. Other tested methods behaves like our method in this regard. However, the performance of KNNimpute degrades much slower than that of LLSimpute and ILLSimpute, which causes that the ensemble method is more sensitive to noise than KNNimpute. Particularly, our method obtains the lowest NRMSE at each noise level. These reflect that the robust to noise is improved by combining multiple individual imputation methods.

### Test results on another dataset

**Fig. 8:**
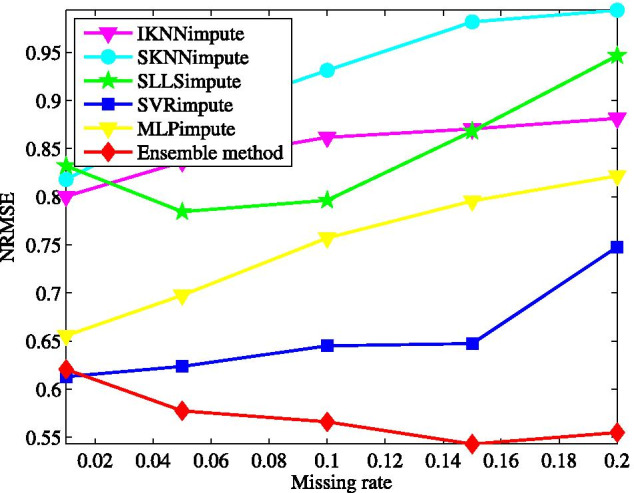
Average NRMSE by SKNNimpute [18], IKNNimpute [19], SLLSimpute [18], SVRimpute [27], MLPimpute [30], and ensemble method on GDS38 dataset with different missing rates

Simulations are also done on another subset of the cancer genomic atlas database [43], called TCGA subset2. Like the previous one, the data matrix contains 50 subjects with 104 microRNAs. Simulation scheme and parameter setting remain the same as before. The obtained NRMSE by each tested imputation method is plotted in Figs. 5, 6 and 7 as a function of missing rate, sample size and noise level, respectively.

In principle, on the second data matrix, the tested imputation methods perform worse than on the previous one in terms of the imputation accuracy. Moreover, among the individual imputation methods, the relative performance depends on the utilized datasets. For example, in Fig. 5, ILLSimpute presents the NRMSE larger than KNNimpute for the missing rate larger than 0.05, which is inconsistent with the observation in Fig. 2. The effect is caused by the fact that neither the global information or the local information remains consistent importance on the imputation for different data matrices. For this reason, it is hard to choose a suitable individual imputation methods in practical applications.

Again, we can see that, in most cases, the proposed method still outperforms other methods significantly despite the change of the data matrix. The good generalization ability is obtained for our method takes use of more data information by integrating diverse imputation methods. Moreover, the weights for each base method are optimized in a data-driven way, by which the importance of the global information or the local information depending on the used dataset can be expressed. This ensures that the ensemble output is best in the sense of Statistics.

### Comparison with other single imputation methods

Furthermore, the proposed ensemble method is compared with SKNNimpute [18], IKNNimpute [19], SLLSimpute [18], SVRimpute [27], and MLPimpute [30]. SKNNimpute and SLLSimpute are respectively the extensions of the basic KNNimpute and LLSimpute with the imputation order for genes considered. IKNNimpute works by iteratively running KNNimpute. SVRimpute explores SVR to predict missing values. And MLPimpute is built based on multilayer perceptron. These methods are chosen for comparison because they are the state-of-the-art imputation techniques for microarray missing data.

Simulations are performed on the data matrix called GDS38 for a study of cell-cycle-regulated genes in Saccharomyces cerevisiae [44]. GDS38 contains 16 subjects with 7680 genes, which were collected at different points in the cell cycle within 7 min. The whole GDS38 is incomplete and has $6.1\%$ missing data. We randomly extract a total of 420 genes without missing data to form the complete data matrix for simulations. The parameters of SKNNimpute, IKNNimpute and SLLSimpute take the values as used in KNNimpute and ILLSimpute before. For SVRimpute, the relaxation variable, the penalty factor and the parameter of radial basis function are set to $10^{-3}$, 1, and 0.033, respectively. They are chosen by a grid search strategy [27]. MLPimpute uses the following parameter settings: the number of inputs and outputs is 16, and the size of hidden layer is 80. The network is trained applying gradient descent with adaptive learning rate and the learning rate is initialize to $10^{-3}$. The training is stopped when the learning rate is less than $10^{-5}$.

The obtained NRMSE for the tested imputation methods is plotted in Fig. 8 as a function of missing rate. From Fig. 8, we observe that, IKNNimpute gets NRMSEs less than SKNNimpute, particularly at a high missing rate. This indicates that the strategy iteratively running KNNimpute is more effective than changing the imputation order. For SLLSimpute, the effect taken by changing the imputation order is also very weak. The two global imputation methods, both SVRimpute and MLPimpute, exhibit the performance advantage over the previous three local methods. Comparing with MLPimpute, SVRimpute has better prediction precision at each tested missing rate. Moreover, SVRimpute is more insensitive to missing rate. Impressively, among all the tested methods, our method yields the superior imputation accuracy at any tested missing rate, and achieves relatively steady performance while missing rate varies within a large range.

### Comparison with hybrid imputation methods

The proposed ensemble method is also compared with several typical hybrid imputation methods including LinCmb [31], BPCA-iLLS [32] and RMI [33]. These methods are chosen for comparison because they are the state-of-the-art hybrid imputation techniques for microarray missing data.

**Fig. 9:**
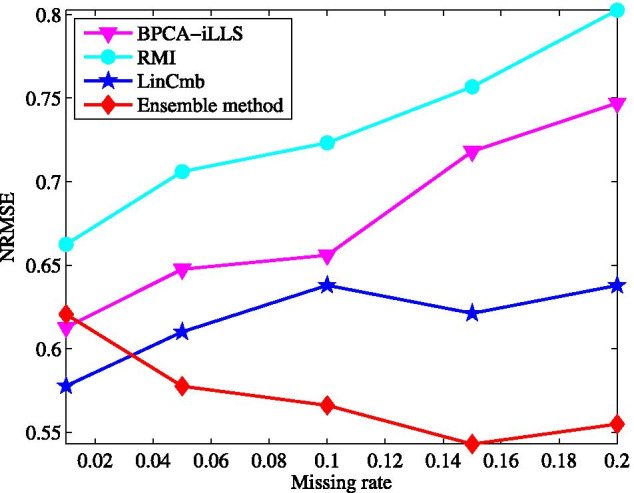
Average NRMSE by LinCmb [31], BPCA-iLLS [32], RMI [33], and ensemble method on GDS38 dataset with different missing rates

The results obtained on GDS38 dataset is shown in Fig. 9, where the default parameter values are taken for LinCmb, BPCA-iLLS, and RMI given in their papers.

Clearly, comparing with the single imputation methods in the last test, all the hybrid methods can obtain much lower NRMSE at the same missing rate, which confirms that the combination of multiple imputation methods is an effective strategy for the imputation performance improvement. Among the existing hybrid methods, LinCmb receives the NRMSE smaller than that of BPCA-iLLS and RMI at each missing rate. Moreover, the performance of LinCmb degrades more smoothly when the missing rate varies from 5 to $20\%$. This is due to the fact that in LinCmb the results of more individual imputation methods are combined into the final prediction. Our method not only has the best imputation accuracy but also possesses the lowest insensitivity to missing rate.

### Effects of the number of base predictors

**Fig. 10:**
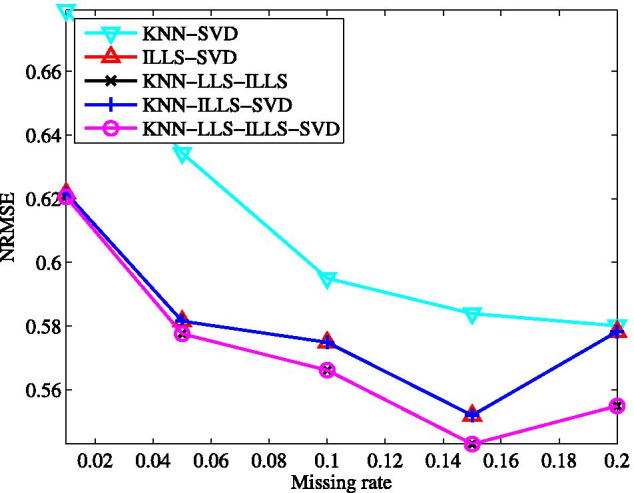
Average NRMSE of ensemble methods combining two to four base predictors respectively on GDS38 dataset with different missing rates

To investigate the effects of the number of base predictors, we vary the number of base predictors in the proposed ensemble method from 2 to 4, and conduct the experiments on GDS38 dataset. The obtained results are shown in Fig. 10 for different missing rates.

As can be seen, among the variants of our full method, the one combining KNNimpute and SVDimpute, called KNN-SVD in Fig. 10 (the names for other ensemble methods in Fig. 10 can be understood in a similar manner) presents the largest NRMSE at each tested missing rate. The combination ILLS-SVD performs better than KNN-SVD, indicating that the performance of the ensemble method largely depends on the used base imputation methods. The combination KNN-ILLS-SVD achieves the imputation performance very close to that of ILLS-SVD, and KNN-LLS-ILLS performs better than the combinations of two base predictors. This shows that the performance of the ensemble method can be improved by increasing the number of the utilized base predictors. Notice that the performance of KNN-LLS-ILLS is very near to one of the ensemble method with four base predictors.

Further, the performance of the variants of our ensemble method (full model) in the test is explained based on the theoretical results given previously. We compute the sample mean $\mu$ and standard deviation $\sigma$ for the base imputation methods and the covariance between each pair of base methods in prediction errors. The symbol $\sigma$ with a subscript represents the covariance between two base methods in prediction errors. For instance, $\sigma _{KL}$ stands for the covariance between KNN and LLS. The results for missing rate $\gamma$ varying from 5 to $15\%$ are summarized in Tables 1 and 2. As is clear, for the case of $\gamma =10\%$, ILLS produces the imputation results with the sample mean in imputation errors close to that of KNN but the standard deviation lower than that of KNN. Hence, according to (19) and (20), the combination ILLS-SVD is theoretically superior to KNN-SVD. Moreover, the performance of ILLS-SVD degrades more gradually with the increase of $\gamma$ because the standard deviation of ILLS is relatively stable for different missing rates.

**Table 1:** Sample mean $\mu$ and standard deviation $\sigma$ for base imputation methods in prediction errors

Method	$\gamma =5\%$		$\gamma =10\%$		$\gamma =15\%$	
	$\mu$	$\sigma$	$\mu$	$\sigma$	$\mu$	$\sigma$
KNN	0.0036	0.4879	0.0114	0.5398	0.0050	0.5760
LLS	0.0089	0.4079	0.0155	0.4385	0.0084	0.4439
ILLS	0.0090	0.4232	0.0158	0.4441	0.0106	0.4606
SVD	0.0038	0.3975	0.0124	0.4185	0.0013	0.4362

The experiment is conducted on GDS38 dataset while missing rate $\gamma$ varies from 5 to $15\%$, and the given sample mean and standard deviation are averaged over the results obtained by repeating the imputation process ten times in bootstrapping way

**Table 2:** Sample covariance for base imputation methods in prediction errors

$\gamma$ (%)	$\sigma _{KL}$	$\sigma _{KI}$	$\sigma _{KS}$	$\sigma _{LI}$	$\sigma _{LS}$	$\sigma _{IS}$
5	0.1160	0.1172	0.0906	0.1589	0.1016	0.1034
10	0.1301	0.1220	0.1057	0.1617	0.1138	0.1126
15	0.1269	0.1312	0.1057	0.1583	0.1179	0.1147

The experiment is conducted on GDS38 dataset while missing rate $\gamma$ varies from 5 to $15\%$, and the given covariance values are averaged over the results obtained by repeating the imputation process ten times in bootstrapping way

Notice that SVD presents the sample mean and standard deviation in imputation errors close to that of LLS. The sample covariance of KNN and LLS in imputation errors is apparently larger than that of KNN and SVD, and the sample covariance of LLS and ILLS in imputation errors is also much larger than that of SVD and ILLS. According to the results and considering (19) and (20), we can analytically derive that KNN-ILLS-SVD should be better than KNN-LLS-ILLS. However, the theoretical analysis is inconsistent with our experimental results. This might be because the combination weights in (4) are learned from the training data other than the testing data. The theoretical performance of the variants with more base predictors is better or not worse than that of the variants with less base predictors. The analytical predictions are in accordance with the experimental results in Fig. 10. Similar observations can be made for other two values of missing rate.

### Application in the classification of tumor samples

Tumor classification is an important application of gene expression data, which is of great significance to the diagnosis and treatment of cancer diseases. In the application, missing data in gene expression data is first imputed and then tumor classification is performed on the imputed data. Therefore, the performance of the imputation method is straightforwardly related to the classification accuracy. In the sequel, we evaluate the performance of the proposed method by conducting a tumor classification experiment.

Tumor cell gene expression data set GDS1761 is used in the experiment, which was sampled from gene expression profile data of 64 cell lines of tumors [45]. This dataset includes breast tumor samples, central nervous system tumor samples, etc., as shown in Table 3, after deleting 3 samples with very much missing data. Each sample is formed by a total of 9706 gene data. The categories, quantities as well as average missing rates of tumor samples are shown in Table 3.

The missing data in the dataset are imputed by applying the ensemble method and other popular imputation methods respectively. After that, the dimensionality of each sample is reduced from 9706 to 16 by the use of principle component analysis (PCA) [46, 47]. Then, gene data are classified into tumor categories listed in Table 3, by carrying out two typical classifiers: *k*-nearest neighbor (KNN) and support vector machine (SVM) [48, 49].

For the performance evaluation method, the leave-one-out cross validation is adopted by considering the small sample number of each tumor category. And the classification performance is assessed by the average accuracy, i.e., the ratio of the number of samples to be correctly classified in all the cross-validation tests to the total times of the cross-validation tests. The experimental results are summarized in Table 4.

**Table 3:** The Number and the missing rate of tumor samples for each tumor category in the used dataset

Tumor	Sample size	Missing rate (%)
Breast tumor	9	15.6
Central nervous system tumors	6	9.1
Colonic neoplasms	7	13.5
Leukemia	8	11.2
Melanoma and birthmark tumors	8	7.6
Non-small cell lung cancer	9	15.7
Ovarian tumor	6	8.5
Kidney neoplasm	8	14.9

**Table 4:** Classification accuracy (%) of KNN and SVM on the used dataset with different imputation methods

Imputation method	KNN (%)	SVM (%)
KNNimpute	72.13	73.77
LLSimpute	72.13	73.77
SVDimpute	72.13	72.13
ILLSimpute	70.49	72.13
IKNNimpute	72.13	73.77
SLLSimpute	72.13	72.13
SKNNimpute	72.13	72.13
ZEROimpute	72.13	72.13
MEANimpute	73.77	75.40
ENSEMBLEimpute	77.05	80.32

Obviously, the classification performance is closely related with the utilized imputation method. A good imputation method helps improve the classification performance. For instance, our ensemble method brings about the significant performance advantage over other tested methods for the two typical classifiers. The behavior indicates that the imputation method with high precision definitely plays a positive role in the subsequent data analysis. However, surprisingly, the simple imputation method, MEANimpute, is more effective than KNNimpute, LLSimpute, SVDimpute and their improved versions in the task of gene data classification. This reveals that the data imputation is less reliable if only exploiting local information (e.g., KNNimpute) or global information (e.g., SVDimpute). In addition, SVM possesses better classification performance than KNN for the same imputation method.

## Conclusion

In this paper, an ensemble method has been proposed for missing value imputation by constructing a set of base imputation methods and combining them. Four commonly used imputation methods served as the base methods and were trained by applying the bootstrap sampling to reduce their dependence. The final predictions were produced by weighting and summing the predictions given by all base methods, where a learning scheme was developed to derive the optimal weights by minimizing the imputation errors with known gene data. Moreover, we theoretically evaluated the performance of the proposed method.

The ensemble imputation method has been extensively tested on several typical genomic datasets, and compared with the state-of-the-art imputation methods including KNNimpute, IKNNimpute, SKNNimpute, LLSimpute, ILLSimpute, SLLSimpute, SVDimpute, SVRimpute, MLPimpute, LinCmb, BPCA-iLLS, and RMI. Experimental results confirmed the advantage of the proposed method over other tested methods consistently in all three different scenarios in terms of lower value of NRMSE. Of particular importance is that our method yields much better generalization and universality.

## Data Availability

The cancer genomic atlas database GDS5669 on Glioma cancer study, the dataset GDS38 for a study of cell-cycle-regulated genes in Saccharomyces cerevisiae, and Tumor cell gene expression dataset GDS1761 are all available at https://www.ncbi.nlm.nih.gov.

## Abbreviations

DNA: Deoxyribonucleic acid
RNA: Ribonucleic acid
mRNA: Messenger ribonucleic acid
KNN: k-Nearest neighbor
KNNimpute: k-Nearest neighbor imputation
IKNNimpute: Iterative KNNimpute
DTW: Dynamic time warping
LLSimpute: Local least square imputation
ILLSimpute: Iterative LLSimpute
SLLSimpute: Sequential LLSimpute
SVDimpute: Singular value decomposition based imputation
BPCA: Bayesian principle component analysis
GBT: Gradient boosted tree
NRMSE: Normalized root mean square error
SVD: Singular value decomposition
miRNA: Micro ribonucleic acid
AWGN: Additive white Gaussian noise
PCA: Principle component analysis
SVM: Support vector machine
KKT: Karush–Kuhn–Tucker
LinCmb: Linear Combination
RMI: Recursive mutual imputation
SVRimpute: Support vector regression for imputation
MLPimpute: The imputation method based on multilayer perceptron networks

## References

[1] Kristensen VN, Kelefiotis D, Kristensen T, Borresen-Dale A-L (2001). High-throughput methods for detection of genetic variation. Biotechniques.

[2] Muro S, Takemasa I, Oba S, Matoba R, Ueno N, Maruyama C, Yamashita R, Sekimoto M, Yamamoto H, Nakamori S, Monden M, Ishii S, Kato K (2003). Identification of expressed genes linked to malignancy of human colorectal carcinoma by parameteric clustering of quantitative expression data. Genome Biol.

[3] Mirus JE, Zhang Y, Li CI, Lokshin AE, Prentice RL, Hingorani SR, Lampe PD (2015). Cross-species antibody microarray interrogation identifies a 3-protein panel of plasma biomarkers for early diagnosis of pancreas cancer. Clin Cancer Res.

[4] Wang W, Iyer NG, Tay HT, Wu Y, Lim TK, Zheng L, Song IC, Kwoh CK, Huynh H, Tan PO (2015). Microarray profiling shows distinct differences between primary tumors and commonly used preclinical models in hepatocellular carcinoma. BMC Cancer.

[5] Shipp MA, Ross KN, Tamayo P, Weng AP, Kutok JL, Aguiar RCT, Gaasenbeek M, Angelo M, Reich M, Pinkus GS, Ray TS, Koval MA, Last KW, Norton A, Lister TA, Mesirov J, Neuberg DS, Lander ES, Aster JC, Golub TR (2002). Diffuse large b-cell lymphoma outcome prediction by gene-expression profiling and supervised machine learning. Nat Med.

[6] Chai LE, Law CK, Mohamad MS, Chong CK, Choon YW, Deris S, Illias RM (2014). Investigating the effects of imputation methods for modelling gene networks using a dynamic Bayesian network from gene expression data. Malays J Med Sci.

[7] Zhang W, Spector TD, Deloukas P, Bell JT, Engelhardt BE (2015). Predicting genome-wide DNA methylation using methylation marks, genomic position, and DNA regulatory elements. Genome Biol.

[8] Wang A, An N, Chen G, Li L, Alterovitz G (2015). Improving PLS-RFE based gene selection for microarray data classification. Comput Biol Med.

[9] Lenz M, Müller F-J, Zenke M, Schuppert A (2016). Principal components analysis and the reported low intrinsic dimensionality of gene expression microarray data. Sci Rep.

[10] Troyanskaya O, Cantor M, Sherlock G, Brown P, Hastie T, Tibshirani R, Botstein D, Altman RB (2001). Missing value estimation methods for DNA microarrays. Bioinformatics.

[11] Arbeitman MN, Furlong EEM, Imam F, Johnson E, Null BH, Baker BS (2002). Gene expression during the life cycle of drosophila melanogaster. Science.

[12] Albrecht D, Kniemeyer O, Brakhage AA, Guthke R (2010). Missing values in gelbased proteomics. Proteomics.

[13] Liew A-C, Law N-F, Yan H (2010). Missing value imputation for gene expression data: computational techniques to recover missing data from available information. Brief Bioinform.

[14] Echelpoel WV, Goethals PLM (2018). Variable importance for sustaining macrophyte presence via random forests: data imputation and model settings. Sci Rep.

[15] Lin W-C, Tsai C-F (2020). Missing value imputation: a review and analysis of the literature (2006–2017). Artif Intell Rev.

[16] Bertsimas D, Pawlowski C, Zhuo YD (2018). From predictive methods to missing data imputation: an optimization approach. J Mach Learn Res.

[17] Little R, Rubin D (1987). Statistical analysis with missing data.

[18] Zhang X, Song X, Wang H, Zhang H (2008). Sequential local least squares imputation estimating missing value of microarray data. Comput Biol Med.

[19] Brás LP, Menezes JC (2007). Improving cluster-based missing value estimation of DNA microarray data. Biomol Eng.

[20] Hsu H-H, Yang AC, Lu M-D (2011). KNN-DTW based missing value imputation for microarray time series data. J Comput.

[21] Kim H, Golub GH, Park H (2005). Missing value estimation for DNA microarray gene expression data: local least squares imputation. Bioinformatics.

[22] Cai Z, Heydari M, Lin G (2006). Iterated local least squares microarray missing value imputation. J Bioinform Comput Biol.

[23] Xiang Q, Dai X, Deng Y, He C, Wang J, Feng J, Dai Z (2008). Missing value imputation for microarray gene expression data using histone acetylation information. BMC Bioinform.

[24] Nikfalazar S, Yeh C-H, Bedingfield S, Khorshidi HA (2020). Missing data imputation using decision trees and fuzzy clustering with iterative learning. Knowl Inf Syst.

[25] Oba S, Sato M-A, Takemasa I, Monden M, Matsubara K-I, Ishii S (2003). A Bayesian missing value estimation method for gene expression profile data. Bioinformatics.

[26] Chen Y, Wang A, Ding H, Que X, Li Y, An N, Jiang L (2016). A global learning with local preservation method for microarray data imputation. Comput Biol Med.

[27] Wang X, Li A, Jiang Z, Feng H (2006). Missing value estimation for DNA microarray gene expression data by support vector regression imputation and orthogonal coding scheme. BMC Bioinform.

[28] Yang M.Q, Weissman S.M., Yang W, Zhang JCA, Guan R (2018). MISC: missing imputation for single-cell RNA sequencing data. BMC Syst Biol.

[29] Yrak TB, Ogul H. Microarray missing data imputation using regression. In: Proceedings of IASTED international conference on biomedical engineering (BioMed), 2017. p. 68–73.

[30] Silva-Ramírez E-L, Pino-Mejía R, López-Coello M, Cubiles-de-la-Vega M-D (2011). Missing value imputation on missing completely at random data using multilayer perceptrons. Neural Netw.

[31] Jönsten R, Wang HY, Welsh WJ, Ouyang M (2005). DNA microarray data imputation and significance analysis of differential expression. Bioinformatics.

[32] Shi F, Zhang D, Chen J, Karimi HR (2013). Missing value estimation for microarray data by Bayesian principal component analysis and iterative local least squares. Math Probl Eng.

[33] Li H, Zhao C, Shao F, Li GZ, Wang X (2015). A hybrid imputation approach for microarray missing value estimation. BMC Genomics.

[34] Nie L, Wu G, Brockman FJ, Zhang W (2006). Integrated analysis of transcriptomic and proteomic data of *Desulfovibrio vulgaris*: zero-inflated Poisson regression models to predict abundance of undetected proteins. Bioinformatics.

[35] Torres-García W, Brown SD, Johnson RH, Zhang W, Runger GC, Meldrum DR (2011). Integrative analysis of transcriptomic and proteomic data of Shewanella oneidensis: missing value imputation using temporal datasets. Mol BioSyst.

[36] Li F, Nie L, Wu G, Qiao J, Zhang W (2011). Prediction and characterization of missing proteomic data in *Desulfovibrio vulgaris*. Comput Funct Genomics.

[37] Lin D, Zhang J, Li J, Xu C, Deng H-W, Wang Y-P (2016). An integrative imputation method based on multi-omics datasets. BMC Bioinform.

[38] Hu J, Li H, Waterman MS, Zhou XJ (2006). Integrative missing value estimation for microarray data. BMC Bioinform.

[39] Jörnsten R, Ouyang M, Wang H-Y (2007). A meta-data based method for DNA microarray imputation. BMC Bioinform.

[40] Yang Y, Xu Z, Song D (2016). Missing value imputation for microrna expression data by using a go-based similarity measure. BMC Bioinform.

[41] Zhou Z-H (2012). Ensemble methods foundations and algorithms.

[42] Hastie T, Tibshirani R, Friedman J (2016). The elements of statistical learning: data mining, inference, and prediction.

[43] The cancer genomic atlas database GDS5669 on Glioma cancer study. https://www.ncbi.nlm.nih.gov. Accessed 20 May 2018.

[44] The data matrix GDS38 for a study of cell-cycle-regulated genes in Saccharomyces cerevisiae. https://www.ncbi.nlm.nih.gov. Accessed 20 May 2018.

[45] Tumor cell gene expression data set GDS1761. https://www.ncbi.nlm.nih.gov. Accessed 20 May 2018.

[46] Li Z-W, Cai X-F, Wei J, Zhou Y (2014). Classification of cancer gene expression profile based on PCA and LDA. Beijing Biomed Eng.

[47] Wang Q-Z, Wang N, Song H, Bao W-X (2014). Identification of cancer-related differentially expressed genes based on entropy measure and PCA. J Biol.

[48] Yu Z, Li T, Wu S (2003). Partial least squares and support vector machine applied to the classification of microarray gene expression data. Comput Appl Chem.

[49] He A, Zhu Y, An K (2007). Tumor molecular classification based on genetic algorithms and support vector machines. J Data Acquis Process.

